# The Effects of Resistance Training on Muscular Fitness, Muscle Morphology, and Body Composition in Elite Female Athletes: A Systematic Review

**DOI:** 10.1007/s40279-023-01859-4

**Published:** 2023-06-08

**Authors:** Amira Zouita, Manel Darragi, Mariem Bousselmi, Zouita Sghaeir, Cain C. T. Clark, Anthony C. Hackney, Urs Granacher, Hassane Zouhal

**Affiliations:** 1 Higher Institute of Sport and Physical Education of Ksar-Said, Research Unit “Sports Performance, Health & Society” (UR17JS01), University of Manouba, Manouba, Tunisia; 2 Higher Institute of Sport and Physical Education of Ksar-Said, University of Manouba, Manouba, Tunisia; 3 Centre for Intelligent Healthcare, Coventry University, Coventry, UK; 4 Department of Exercise & Sport Science, University of North Carolina, Chapel Hill, NC USA; 5 Department of Sport and Sport Science, Exercise and Human Movement Science, University of Freiburg, Freiburg, Germany; 6 Univ Rennes, M2S (Laboratoire Mouvement, Sport, Santé), EA 1274, 35000 Rennes, France

## Abstract

**Background:**

Well programmed strength and conditioning training is an indispensable part of the long-term training process for athletes in individual and team sports to improve performance and prevent injuries. Yet, there is a limited number of studies available that examine the effects of resistance training (RT) on muscular fitness and physiological adaptations in elite female athletes.

**Objectives:**

This systematic review aimed to summarize recent evidence on the long-term effects of RT or combinations of RT with other strength-dominated exercise types on muscular fitness, muscle morphology, and body composition in female elite athletes.

**Materials and Methods:**

A systematic literature search was conducted in nine electronic databases (Academic Search Elite, CINAHL, ERIC, Open Access Theses and Dissertations, Open Dissertations, PsycINFO, PubMed/MEDLINE, Scopus, and SPORTDiscus) from inception until March 2022. Key search terms from the MeSH database such as RT and strength training were included and combined using the operators “AND,” “OR,” and “NOT”. The search syntax initially identified 181 records. After screening for titles, abstracts, and full texts, 33 studies remained that examined the long-term effects of RT or combinations of RT with other strength-dominated exercise types on muscular fitness, muscle morphology, and body composition in female elite athletes.

**Results:**

Twenty-four studies used single-mode RT or plyometric training and nine studies investigated the effects of combined training programs such as resistance with plyometric or agility training, resistance and speed training, and resistance and power training. The training duration lasted at least 4 weeks, but most studies used ~ 12 weeks. Studies were generally classified as ‘high-quality’ with a mean PEDro score of 6.8 (median 7). Irrespective of the type or combination of RT with other strength-dominated exercise regimens (type of exercise, exercise duration, or intensity), 24 out of 33 studies reported increases in muscle power (e.g., maximal and mean power; effect size [ES]: 0.23 < Cohen’s *d* < 1.83, small to large), strength (e.g., one-repetition-maximum [1RM]; ES: 0.15 < *d* < 6.80, small to very large), speed (e.g., sprint times; ES: 0.01 < *d* < 1.26, small to large), and jump performance (e.g., countermovement/squat jump; ES: 0.02 < *d* < 1.04, small to large).

The nine studies that examined the effects of combined training showed significant increases on maximal strength (ES: 0.08 < *d* < 2.41, small to very large), muscle power (ES: 0.08 < *d* < 2.41, small to very large), jump and sprint performance (ES: 0.08 < *d* < 2.41, small to very large). Four out of six studies observed no changes in body mass or percentage of body fat after resistance or plyometric training or combined training (ES: 0.026 < d < 0.492, small to medium). Five out of six studies observed significant changes in muscle morphology (e.g., muscle thickness, muscle fiber cross-sectional area; ES: 0.23 < *d* < 3.21, small to very large). However, one study did not find any changes in muscle morphology (i.e., muscle thickness, pennation angle; ES: 0.1 < *d* < 0.19, small).

**Conclusion:**

Findings from this systematic review suggest that RT or combined RT with other strength-dominated exercise types leads to significant increases in measures of muscle power, strength, speed, and jump performance in elite female athletes. However, the optimal dosage of programming parameters such as training intensity and duration necessary to induce large effects in measures of muscular fitness and their physiological adaptations remain to be resolved in female elite athletes.

## Key Points

**Table:** 

In highly trained female athletes, RT or combinations of RT with other strength-dominated exercise regimens lead to significant increases in power (e.g., maximal and mean power), strength (e.g., 1RM), speed (e.g., linear sprint times), and jump performance (e.g., countermovement jump), irrespective of the applied programming parameters (type of exercise, exercise duration or intensity).
Discrepancies remain concerning the effects of RT or combinations of RT with other strength-dominated exercise regimens on muscle morphology (e.g., muscle thickness, muscle fiber cross-sectional area) in highly trained female athletes.
The optimal dosage of training intensity and duration necessary to produce the most effective adaptations remain unclear in this population.

## Introduction

Success in modern sports is the ultimate goal for both athletes and coaches. Adequate programming of resistance training (RT) regimens is needed complementary to sport-specific training to develop the physical fitness and athletic performance of youth and elite athletes [1, 2]. During long-term athlete development, there should be specific focus on the promotion of muscular fitness, irrespective of the individual’s maturational status and sex [3]. Sufficient levels of muscular fitness are the foundation for motor skill learning and enable athletes to tolerate the demands of long-term training and competition [4, 5]. Smith and colleagues [6] introduced muscular fitness as an umbrella term for muscle strength, muscle power, and local muscular endurance. The effectiveness of various RT types (e.g., free-weight training, machine-based training, plyometric) on measures of physical fitness and sport-specific performance is well established for youth [5, 7–9] and elite athletes [10, 11]. However, most of the available research has been conducted with males and less with females [12, 13].

Currently, most systematic reviews of RT variables have been performed only on male or mixed-sex samples [10, 14]. Hagstrom et al. [15] stated that there were no reviews conducted specifically for females, so the main purpose of their review was to quantify the effects of RT in females and summarize the existing literature by gender. Because males and females differ in circulating anabolic hormones (e.g., testosterone), anatomy (e.g., limb length and pelvic angles, muscle size, and body composition), and physiology (e.g., cardiorespiratory fitness, metabolic factors, fatigability, and inflammatory responses), there may be differences in how females adapt to RT [16].

The review by Hagstrom et al. [15] provided evidence-based estimates of women's fitness for RT. In their review, an average lean body mass gain of 3.3% was observed, which equates to approximately 1.45 kg (range 0.4–3.3 kg) following a full body program. Muscle strength increased approximately 25% (range 4–40%) in the upper body and 27% (range 6.5–54%) in the lower body. These adjustments occurred after participation in a program with an average duration of 15 weeks. Typically, prescribed parameters include a frequency of three training sessions per week and three sets of approximately ten repetitions per exercise. When intensity is expressed as a percentage of 1RM, the average exercise intensity was 70%.

In the form of original research, Montalvo-Pérez et al. [13] examined the effects of 6 weeks of short-term velocity-based RT compared with traditional RT (i.e., three sets per exercise interspersed by 120-s rest periods, with intensity progressively increasing from 80 to 90% of 1RM from the start to the end of the intervention period) on body composition, muscle strength/power, and endurance in competitive female cyclists. The results showed marked improvements in muscle strength/power as well as a slight increase in time trial performance (∼3–5%), with no differences between interventions but with short-term velocity-based RT inducing greater increases in maximum strength/power for the hip thrust exercise [13]. Moreover, Lesinski et al. [7] examined the seasonal effects of strength endurance training versus power training on measures of physical fitness and body composition in young female soccer players. While strength endurance training showed significantly better ventral core strength (plank test) and change-of-direction performance (T-test), power training resulted in significantly better 1RM leg press, vertical jump, and linear sprint performances. The authors concluded that strength endurance training and power training complement each other and can be implemented in young female soccer players.

Using a meta-analytical approach, Moran and colleagues [8] described the effects of RT on muscle strength in female youth. Based on 11 included studies, the authors reported small overall effects. Effect sizes were larger in girls aged > 15 years. In another meta-analysis, the same authors [9] examined the effects of plyometric training (jump training) on measures of muscle strength in youth females. Fourteen studies were included, and the authors showed overall small training effects for vertical jump performance. For plyometric training, effect sizes were larger for girls aged < 15 years. While these studies provide helpful but preliminary information on the effectiveness of RT with (youth) females, the main body of strength and conditioning research focuses on males (athletes) [10, 16].

When systematic reviews on RT were performed in female cohorts only, they primarily dealt with clinical outcomes such as breast cancer lymphedema [10] or bone mineral density [17]. Not surprisingly, the main finding of the review performed by Hagstrom et al. [15] was that RT had a significant effect on muscle strength and hypertrophy in untrained healthy adult women. However, the literature included in the meta-analysis was of moderate quality [15]. Therefore, a review of the existing but limited literature on the effects of RT on muscular fitness and sport-specific performance in women seems timely and necessary, especially for elite female athletes.

## Methods

### Search Strategy

This systematic review was conducted in accordance with the Preferred Reporting Items for Systematic Reviews and Meta-Analyses (PRISMA) Statement [18] and the Cochrane Handbook for Systematic Reviews of Interventions [19]. The study protocol was registered in the Open Science Framework (OSF) platform (https://doi.org/10.17605/OSF.IO/83KD6). The PICOS approach (Population, Intervention, Comparator, Outcomes, Study design) was followed to identify inclusion criteria (Table 1).

**Table 1:** Inclusion criteria according to the PICOS approach

PICOS components	Details
Population	Well trained, elite female athletes, i.e., highly trained athletes training at least 4 times per week and competing at the national or international level in their sport
Intervention	Long-term RT studies or combinations of RT with other strength-dominated exercise types
Comparator	Active and/or passive controls
Outcomes	Measures of muscle strength (e.g., 1-repetition maximum [1RM], maximum voluntary contraction), proxies of muscle power (e.g., vertical and horizontal jump performance), muscle morphology (e.g., muscle mass, muscle size, muscle fiber), and body composition (e.g., body mass, body fat, lean mass)
Study design	nRCTs, nRnCTs, and RCTs

*nRCT* non-randomized controlled trial, *nRnCT* non-randomized non-controlled trial, *RCT* randomized controlled trial

### Eligibility Criteria

Only randomized controlled trials and controlled trials that examined the long-term effects of RT or combinations of RT with other strength-dominated exercise types on muscle strength and power, muscle morphology, and body composition in highly trained female athletes were eligible for inclusion. Studies were included in the current systematic review if they were in accordance with the following criteria: (i) published in peer-reviewed journals; (ii) included elite female athletes and highly trained women according to the definition of McKay et al. [20] (i.e., highly trained athletes that exercise at least four times per week and compete at national or international level in their sport); (iii) used validated methods of monitoring training load; (iv) used RT or combinations of RT with other strength-dominated exercise types lasting a minimum of 4 weeks; (v) applied measures of muscular fitness or muscle morphology as outcome parameters. Studies were excluded if they (i) did not meet the minimum requirements of an experimental study design (e.g., case reports); (ii) did not meet the minimum requirements regarding study design (e.g., lack of information on training methodology or testing sessions); (iii) did not apply RT or combinations of RT with other strength-dominated exercise types; (iv) were not written in English; (v) involved untrained participants; or (vi) included male participants. Moreover, review articles of any type were not included in the current systematic review.

### Literature Search Strategy

This systematic literature search was conducted in nine electronic databases: Academic Search Elite, CINAHL, ERIC, Open Access Theses and Dissertations, Open Dissertations, PsycINFO, PubMed/MEDLINE, Scopus, and SPORTDiscus, from database inception until March 2022. The following key terms (and synonyms searched for in the MeSH database) were included and combined using the operators “AND”, “OR”, “NOT”: ((“strength training” OR “resistance training” OR “plyometric training” OR plyometrics OR “resistance exercise” OR “weight lifting” OR weightlifting OR “strength exercise” OR “weight-bearing exercise” OR “resistive exercise” OR “resistive training”) AND (“muscular fitness” OR “muscle fitness” OR “muscle strength” OR “muscular strength” OR strength OR “muscle power” OR “muscular power” OR power OR “muscular endurance” OR “muscle endurance” OR “local muscular endurance” OR endurance) AND (“muscle hypertrophy” OR “muscular hypertrophy” OR “muscle mass” OR “muscle fiber” OR “muscle size” OR “muscle fibre” OR “muscle thickness” OR “cross sectional area” OR “computed tomography” OR “magnetic resonance imaging” OR “pennation angle”) AND (“lean body mass” OR “fat-free mass” OR “body mass index” OR “BMI”) AND (“trained women” OR “trained female” OR “elite female athletes” OR “elite women athletes”)). In addition, the reference lists and citations (Google Scholar) of the identified studies were explored in order to detect further relevant research papers. Since the scope of this review article is broad in terms of outcome measures (e.g., muscle strength, muscle power, muscle morphology, and body composition), a systematic review and not a meta-analysis were performed since a large number of outcome parameters would have produced substantial heterogeneity.

### Working Definitions

- RT is a collective term that refers to methods of physical conditioning that involve the progressive use of a wide range of resistive loads, different movement velocities, and a variety of training modalities [21].
- Plyometric training consists of quick, powerful actions that involve muscle lengthening, immediately followed by rapid shortening of the same muscle in the stretch–shortening cycle [22].
- Muscular strength can be defined as the maximal force or tension a muscle or a group of muscles can generate at a specified velocity [5].
- Elite female athletes refer to well-trained athletes, i.e., highly trained athletes that trained at least four times per week and competed at the national or international level in their sport [20].

### Study Selection and Data Extraction

The final screening was done by two investigators (AZ and MD) based on the relevance of the inclusion and exclusion criteria and the identified items for assessing the long-term effects of RT on muscular strength and power, muscle morphology, and body composition in female elite athletes using PICOS criteria. If the title showed any potential relevance, it was screened at the abstract level. When abstracts indicated potential inclusion, full-text articles were reviewed. Furthermore, the researchers independently analyzed the full texts and determined the eligibility of the studies, and disagreements were resolved by consensus. The agreement rate between reviewers was 93% for the eligibility criteria of the study. A third-party consensus meeting was held with a third author (HZ) if the two reviewers were not able to reach an agreement on the inclusion of an article.

### Assessment of Risk of Bias and Quality Assessment

The methodological quality of the included studies was assessed using the Physiotherapy Evidence Database (PEDro) scale (https://PEDro.org.au/), which has been shown to have good reliability and validity [23–25]. The PEDro scale has 11 possible points that examine external validity (criterion 1) and internal validity (criteria 2–9) of controlled trials and whether there is sufficient statistical information for interpreting results (criteria 10–11). The items of the scale are (i) eligibility criteria were specified; (ii) subjects were randomly allocated to groups; (iii) allocation was concealed; (iv) groups were similar at baseline; (v) subjects were blinded; (vi) therapists who administered the treatment were blinded; (vii) assessors were blinded; (viii) measures of key outcomes were obtained from more than 85% of subjects; (ix) data were analyzed by intention to treat; (x) statistical comparisons between groups were conducted; and (xi) point measures and measures of variability were provided. The first criterion is not included in the final score. Moreover, because of the nature of physical activity interventions, patient and therapy blinding and allocation is unlikely, therefore the total score a trial could receive was 8 points. A cut-off point of 6 on the PEDro scale was used to indicate high-quality studies, as this has been reported to be sufficient to determine high quality versus low quality in previous studies [25]. Two independent researchers (AZ and MD) assessed the quality of the studies; if any ambiguity arose, a third researcher (HZ) was contacted and a unanimous decision was achieved.

## Data Analysis

Absolute changes (final value versus initial value) in performance indicators of RT on muscular strength and power, muscle morphology, and body composition in female elite athletes were reported as differences between arithmetic means before and after the interventions. The results of intention-to-treat analyses were always used when available in the selected studies. Calculations were performed using a random-effects model. The results of the exercising groups were compared with the results of the control group (non-exercise). For all variables, in the comparisons between exercising groups (e.g., two or more training modes), the studies were stratified by considering the presence of equalization. A study was considered equalized when the design was adopted for different training programs with similar energy expenditures or similar workloads. This information should be mentioned in the article or presented in the results. When this information was not presented, the study was classified as non-equalized.

The level of significance was set at 5%. For statistical heterogeneity of the treatment effect between the studies, a threshold *p-*value of 0.1 estimated by the Cochran *Q* test was rated as statistically significant. For heterogeneity, values > 50% in the inconsistency *I*^2^ test were considered indicative of high heterogeneity. Because some studies had more than one training group with a single control group, this shared control group was divided into two or more groups with smaller sample sizes and was weighted in relation to the different exercise interventions. This procedure was performed to obtain independent comparisons and overcome a unit-of-analysis error for studies that could contribute to multiple and correlated comparisons, as suggested by the Cochrane Handbook for Systematic Reviews of Interventions [19].

Transformation methods were used for studies that presented results as the standard error of the mean, confidence intervals, or interquartile ranges [26]. Data not available and not made available by the corresponding author were imputed. In those situations, the weighted average of all available studies for the variable in question was considered. To conduct the multiple comparisons (different exercising groups vs control), a network model was adopted. For this, the weighted average of all available studies was considered for group imputation.

To strengthen the robustness of the findings, sensitivity analyses were performed by deleting each study separately to analyze the influence of each study on the overall results. All analyses were performed using REVIEW MANAGER software, version 5.3 (Cochrane Collaboration, London, UK).

The percent change (Δ%) was calculated (if not available in the study) for each study to evaluate the magnitude of the effects using the following equation:

$$\Delta \% = \left( {M_{{{\text{post}}}} - M_{{{\text{pre}}}} } \right)/M_{{{\text{pre}}}} \times 100.$$

where *M*_post_ represents the mean value after long-term training and *M*_pre_ the baseline mean value.

Effect size (ES) was computed to present standardized effects of long-term training on the outcome variables (e.g., strength performances, muscle power, muscle morphology, and body composition). The ES was calculated with Cohen’s d [27, 28], by dividing the raw ES (difference in means) by the pooled standard deviations:

$${\text{ES}} = \left( {{\text{Cohen}}{}^{^{\prime}}{\text{s}}\;d} \right) = \left. {\left( {M_{1} - M_{2} } \right)/{\text{SD}}\;{\text{pooled}}} \right).$$

Values for ES were defined as trivial (< 0.2), small (0.2–0.6), moderate (0.6–1.2), large (1.2–2.0), and very large > 2 [23, 29]. Results for each outcome variable are presented with the number of observations (N), Δ%, and ES. Data analyses were processed using SigmaStat 3.5 software (Systat, Inc, USA). The ES and Δ% were analyzed in studies where sufficient data were available.

## Results

### Study Selection

**Fig. 1:**
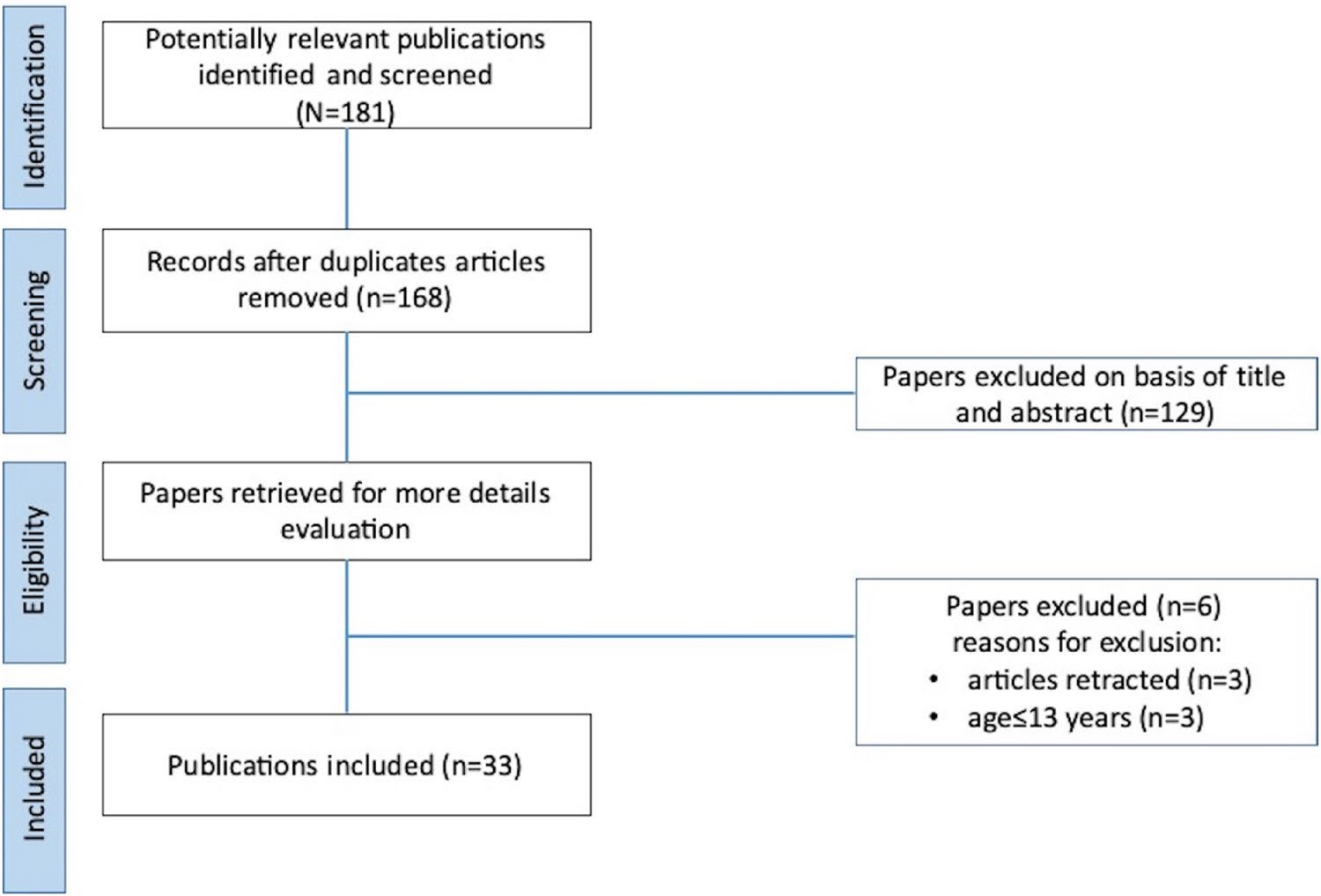
Flow diagram for the selection of studies. The selection process for research articles (*n* = 33) included in this systematic review is an adapted version of the recommendations in the PRISMA (Preferred Reporting Items for Systematic Reviews and Meta-Analyses) statement [88]

Our search identified 181 studies related to the effects of long-term RT or combinations of RT with other strength-dominated exercise types on muscular strength and power, muscle morphology, and body composition in female elite athletes (Fig. 1). After the screening of titles, abstracts, and full texts, 33 studies were selected for inclusion in our final analysis, and the characteristics of these long-term studies are summarized in Table 2. The 33 studies were carried out in different countries from five continents (Africa, North America, Europe, Asia, and Australia).

**Table 2:** Characteristics of the studies that examined the long-term effects of RT (a), plyometric training (b), or combinations of RT (c), with other strength-dominated exercise types on muscular fitness, body composition, and muscle morphology in elite female athletes

(a) Resistance training											
Study characteristics			Participant characteristics				Resistance training protocol				
Study	PEDro scale	Country	*N* IG	*N* CG	Age, years (mean ± SD or range)	Sport	Exercise type	Duration (weeks)	Frequency per week	Intensity	Volume
Ness et al. [49]	6	USA	17	no CG	18.8 ± 0.9	Soccer	Speed strength	8	3		60 min per session
							Hang clean		3		Set 4–6 / reps 2–3
							Barbell deadlift		2		Set 4–8 / 3–5 reps
							Push up		3		Set 4–8 / reps 2–1
							Back squat		2		set 4–8 / reps 2–8
							Single arm row		2		Set 3–4 / reps 5–12
Losnegard et al. [47]	7	Norway	3	5	21.3 ± 5.1	Cross-country skiing		12	2		45 min each session
Jones et al. [51]	3	USA	28	no CG	Not specified	Soccer	Lower-body push	12	3		2 whole-body lifting sessions 8–10 reps 6–8 reps 3–4 exercises / 1–3 sets of 10–15 reps
			28		Not specified	Field hockey	Upper-body pull				
			19		Not specified	Softball	Circuit: abs/low back				
Montalvo-Pérez et al. [13]	8	Spain	TRT *n* = 8	no CG	26 ± 7	Cycling		6	2	80–90% of 1RM	3 sets / exercise 120-s rest periods 4–8 reps
			VBRT *n* = 9	no CG			Velocity-based RT		2		3 sets / exercise 120-s rest periods
Newton et al. [50]	7	Australia	14	no CG	20.0 ± 1.2	Volleyball	8–11 weeks: loaded jump squat performed ballistically on the Smith machine	11	2	Heavy ballistic training	3 sets of 6 reps 3 sets of 3 reps
Shalfawi et al. [52]	6	Norway		no CG	19.4 ± 4.4	Soccer	Leg press	10	2	10RM	2 3 2 × 6–3 × 4 2 3
							SJ			4RM	
							Cable hip flexion			10RM	
							Cable hip extension			4RM	
Skattebo et al. [43]	6	Norway	9	7	18 ± 1	Cross-country skiing	Seated pull-down	10	2	Sub-maximal 40% RM 75% RM 85% RM	3 sets 10 reps 6 reps 3 reps 2–3 min rest periods
Vikmoen et al. [48]	8	Norway	11	8	Not specified	Athletics	4 leg exercises: half squat in a smith machine leg press with one leg at a time standing one-legged hip flexion ankle plantar flexion	11	2	*Weeks 1–3* 1st session: 10RM sets 2nd session: 6RM sets *Weeks 4–6* 1st session: 8RM 2nd session: 5RM sets *Weeks 7–11* 1st session: 6RM 2nd session: 4RM	3 × 4 exercises 10 reps
Vikmoen et al. [11]	8	Norway	11	8	Not specified	Athletics	4 leg exercises: half squat in a Smith machine leg press with one leg at a time standing one-legged hip flexion ankle plantar flexion	11	Twice a week	*Weeks 1–3* 1st session: 10RM sets 2nd session: 6RM sets *Weeks 4–6* 1st session: 8RM 2nd session: 5RM sets *Weeks 7–11* 1st session: 6RM 2nd session: 4RM	3 sets/exercises 10 reps
Vikmoen et al. [42]	6	Norway	11	10	31.5 ± 8.0	Athletics	4 leg exercises: half squat in a Smith machine leg press with one leg at a time standing one-legged hip flexion ankle plantar flexion	11	2	3 weeks, 10RM sets at the first session and 6-RM sets at the second session of the week *Weeks 4–6* 8RM and 5RM *Weeks 7–11* 6RM and 4RM	3 × 4 exercises × 10 reps

(b) Plyometric training										
Study characteristics			Participant characteristics				Plyometric training protocol			
Study	PEDro scale	Country	N IG	N CG	Age, years (mean ± SD or range)	Sport	Duration (weeks)	Frequency per week	Intensity	Volume
Exercise type: plyometric training (strength + power), example of exercise: bouncing jump, hurdle jump, drop, lateral cone jumps, tuck jump, aquatic plyometric training										
Attene et al. [60]	8	Italy	18	18	14.9 ± 0.9	Basketball	6	2		20 min 5 exercises
Chimera et al. [46]	7	USA	9	9	20 ± 2	Soccer	6	2		20–30 min each session
Fischetti et al. [63]	9	Italy	14	14	26.5 ± 6.9	Soccer	12	3		40–60 min 5–20 / 5–30 reps
Krističević et al. [62]	6	Croatia	54	no CG	15.4 ± 1.32	Volleyball	5			
Maciejczyk et al. [30]	7	Poland	17	8	21 ± 3	Soccer	4	2		8 sessions
Myer et al. [64]	8		8	no CG	15.9 ± 0.8	Basketball Soccer Swimming Softball	7	3	Max effort	10–15 times 10–15 times 15 times 15 times
Ozbar et al. [61]	7	Turkey	9	9	18.2 ± 2.3 (15–22)	Soccer	8	1	Low impact, low frequency	60 min per session 4–5 sets 5–15 reps of 4–5 exercises
Ozbar [41]	7	Turkey	10	10	19.4 ± 1.6 (18–22)	Soccer	10	2	Low to high	20–30 min 3–5 sets 5–8 reps 6–8 exercises
Pereira et al. [65]	7	Portugal	10	10	14.0	Volleyball	8	2		20 min 3–5 × 20 3–4 × 10
Usman and Shenoy [67]	8	India	30	30	19.2 ± 0.8	Volleyball	8	2	Low to high intensity	
Martel et al. [35]	9	USA	10	9	15 ± 1		6	2		45 min 3–6 times

(c) Combinations of RT with other strength-dominated exercise types (e.g., RT in combination with plyometric training or agility training)											
Study characteristics			Participant characteristics				Protocols of combined RT with other strength-dominated exercise types				
Study	PEDro scale	Country	N IG	N CG	Age, y (mean ± SD or range)	Sport	Exercise type	Duration (weeks)	Frequency per week	Intensity	Volume
Grieco et al. [39]	4	USA	15	no CG	19.0 ± 0.7	Soccer	plyometric and agility training: Combined RT Plyometric-agility training drills	10	2		60 min 9–10 exercises of 3 sets each
Delextrat et al. [31]	8	Colombia	R 10	no CG	21.8 ± 4.0	Soccer	RT: leg curl and stiff-leg deadlift	7		6RM	3–5 sets 3-min inter-set rest
			RE 11		23.7 ± 7.2		RE: leg curl and stiff-leg deadlift			12–20RM	3 sets 45–90 s inter-set rest
Luteberget et al. [32]	4	Norway	10	8	20.4 ± 3.1	Handball	Resisted sprint training	10	2	12.4% ± 0.2% of body mass	
			28		Not specified	Field hockey					
			19		Not specified	Softball					
Marques et al. [34]	5	Portugal	5	no CG	16.8 ± 0.8	Swimming	RT *program:*	20	2 cycles of 9 weeks		Reps per set by each participant progressively increased from 50 to 75%
							SJ (%1RM)			40–60%	
							CMJ			15–25%	
							BP (%1RM)			45–65%	
							Shoulder press (%1RM)			20–35%	
Marques et al. [33]	5	Portugal	10	no CG	25.3 ± 1.3	Volleyball	Resistance and power training: RT BP back squat	12	2	50–80% of (4RM)	50 min, 3 sets of 3–6 repetitions
Martel et al. [35]	9	USA	10	9	15 ± 1		Aquatic plyometric training Power skips, single- and double-leg bounding, SJ with blocking form, and depth jumps Depth-jump circuit	6	2		45 min 3–6 reps 3–4 reps 2–4 reps
Nunes et al. [40]	5	Brazil	12	no CG	26.2 ± 3.9	Basketball	Muscular endurance Resistance Power	7	10–12	Maximum (RM) loads 50% 1RM loads	3–4 sets with 15–20 reps
								3			3–6 sets
								2			1–10RM load reps
								2			3–6 sets of 6–10 reps
Pacholek et al. [36]	5	Slovakia	13	no CG	20.2 ± 3.3	Soccer	Complex (the intermittent load type)	9		30–40% 1RM	1–3 sets undefined
							Combined resistance: maximal resistance method + the dynamic concentric	9	2–3	30–40% 1RM	1–3 sets 15–30 reps
										80% from 1RM (concentric)	3–6 reps
										30% of 1RM	15–30 reps
Siegler et al. [44]	7	USA	17	17	16.5 ± 0.9	Soccer	Plyometric program in-season RT, and high-intensity anaerobic program	10	3		10–15 min
									2	lower-body free weights exercises	30 min, 3 sets of 4
Veliz et al. [37]	7	Spain	11	10	26.4 ± 4.3	Water polo	Lower-body resistance and power-oriented training	16	2		30–45 min each session
Voelzke et al. [38]	9	Germany	8	no CG	26.0 ± 7.0	Volleyball	Resistance + plyometric training	5			10 training sessions 4–5 sessions per week; ∼120 min per session
							EMS + plyometric stimulation period		2	85% of the 1RM	10 min The rise time was 0.75 s the fall time was 0.5 s 5 s of tetanic muscle stimulation
Wilkerson et al. [74]	8	USA	11	8	19 ± 1.4	Basketball	Plyometric jump training	6		3 phases of progressively increasing jump complexity and intensity	

*BP* bench press, *CG* control group, *CMJ* countermovement jump, *EMS* + *P* electromyostimulation and plyometric training, *IG* intervention group, *Max* maximum, *min* minute, *N* sample size, *RE* resistance endurance training, *Reps* repetitions, *RM* the maximum load an individual can lift under standardized conditions, *RT* resistance training, *SJ* squat jump, *TRT* traditional resistance training, *VBRT* velocity-based resistance training

A total of 738 participants aged 14–31 years performed RT and 220 participated in the control groups and completed the studies. Athletes from different sports were involved (athletics, cycling, basketball, handball, volleyball, softball, soccer, field hockey, swimming, cross-country skiing, and water polo), but the most represented sport was soccer, with 13 studies.

The 33 studies used different resistance exercise training protocols, with 24 studies using single-mode RT or plyometric training programs, and nine studies investigating the effects of combined training programs such as RT with plyometric or agility training, RT with speed training, and resistance and power training. Training duration ranged between 5 and 12 weeks with a mean duration of 9.4 weeks. According to the PEDro score, the selected studies were classified as ‘high-quality’ studies with a mean PEDro scale score of 6.8 (median 7) (Table 3).

**Table 3:** Physiotherapy evidence database (PEDro) score of the included longitudinal studies

	Study	Eligibility criteria	Randomized allocation	Blinded allocation	Group homogeneity	Blinded subjects	Blinded therapists	Blinded assessor	Drop out 15%	Intention-to-treat analysis	Between-group comparison	Point estimates and variability	PEDro sum
1	Newton et al. [50]	✔	✔	Ο	✔	Ο	Ο	Ο	✔	✔	✔	✔	7
2	Wilkerson et al. [74]	✔	Ο	✔	✔	✔	Ο	Ο	✔	✔	✔	✔	8
3	Attene et al. [60]	✔	✔	✔	✔	Ο	Ο	Ο	✔	✔	✔	✔	8
4	Ness et al. [49]	✔	Ο	Ο	✔	Ο	Ο	✔	✔	✔	Ο	✔	6
5	Cherni et al. [45]	✔	✔	✔	✔	Ο	Ο	Ο	✔	✔	✔	✔	8
6	Chimera et al. [46]	✔	✔	Ο	✔	Ο	Ο	Ο	✔	✔	✔	✔	7
7	Delextrat et al. [31]	✔	✔	✔	✔	Ο	Ο	Ο	✔	✔	✔	✔	8
8	Fischetti et al. [63]	✔	✔	✔	✔	✔	Ο	Ο	✔	✔	✔	✔	9
9	Nunes et al. [40]	✔	Ο	Ο	✔	Ο	Ο	Ο	✔	✔	Ο	✔	5
10	Losnegard et al. [47]	✔	✔	✔	✔	Ο	Ο	Ο	✔	✔	✔	Ο	7
11	Maciejczyk et al. [30]	✔	✔	Ο	✔	Ο	Ο	Ο	✔	✔	✔	✔	7
12	Jones et al. [51]	✔	Ο	Ο	Ο	Ο	Ο	Ο	Ο	Ο	✔	✔	3
13	Marques et al. [33]	✔	Ο	Ο	Ο	Ο	Ο	Ο	✔	✔	✔	✔	5
14	Marques et al. [34]	✔	Ο	Ο	Ο	✔	Ο	Ο	✔	✔	Ο	✔	5
15	Martel et al. [35]	✔	✔	✔	✔	✔	Ο	Ο	✔	✔	✔	✔	9
16	Montalvo-Pérez et al. [13]	✔	✔	✔	✔	Ο	Ο	Ο	✔	✔	✔	✔	8
17	Myer et al. [64]	✔	✔	Ο	✔	Ο	Ο	Ο	Ο	✔	✔	✔	6
18	Ozbar [41]	✔	✔	✔	✔	Ο	Ο	Ο	✔	✔	✔	Ο	7
19	Pacholek et al. [36]	✔	✔	Ο	✔	Ο	Ο	Ο	Ο	✔	Ο	✔	5
20	Pereira et al. [65]	✔	✔	✔	✔	Ο	Ο	Ο	✔	✔	✔	Ο	7
21	Shalfawi et al. [52]	✔	✔	✔	Ο	Ο	Ο	Ο	Ο	✔	✔	✔	6
22	Usman and Shenoy [67]	✔	✔	✔	✔	Ο	Ο	Ο	✔	✔	✔	✔	8
23	Siegler et al. [44]	✔	Ο	✔	✔	Ο	Ο	Ο	✔	✔	✔	✔	7
24	Skattebo et al. [43]	✔	Ο	✔	Ο	Ο	Ο	Ο	✔	✔	✔	✔	6
25	Krističević et al. [62]	✔	✔	Ο	✔	Ο	Ο	Ο	✔	Ο	✔	✔	6
26	Veliz et al. [37]	✔	✔	Ο	✔	Ο	Ο	Ο	✔	✔	✔	✔	7
27	Vikmoen et al. [48]	✔	✔	✔	✔	Ο	Ο	Ο	Ο	✔	✔	✔	7
28	Vikmoen et al. [42]	✔	✔	✔	✔	Ο	Ο	Ο	✔	✔	✔	✔	8
29	Vikmoen et al. [11]	✔	✔	✔	✔	Ο	Ο	Ο	✔	✔	✔	✔	8
30	Voelzke et al. [38]	✔	✔	✔	✔	Ο	Ο	Ο	✔	✔	✔	✔	8
31	Grieco et al. [39]	✔	Ο	Ο	✔	Ο	Ο	Ο	Ο	✔	Ο	✔	4
32	Luteberget et al. [32]	✔	Ο	Ο	✔	Ο	Ο	Ο	Ο	Ο	✔	✔	4
33	Ozbar et al. [61]	✔	✔	✔	✔	Ο	Ο	Ο	✔	✔	✔	Ο	7

### Effects of Long-Term Resistance Training or Combinations of Resistance Training With Other Strength-Dominated Exercise Types on Muscular Fitness in Elite Female Athletes

**Fig. 2:**
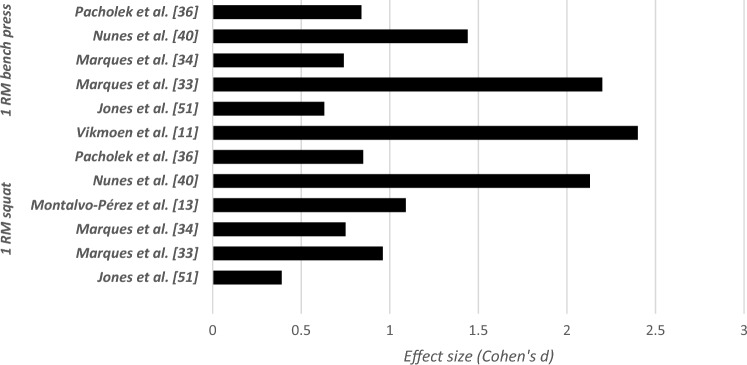
Summary of effect sizes of the identified studies on the effectiveness of RT on one-repetion-maximum (1RM) performance in female elite athletes

**Fig. 3:**
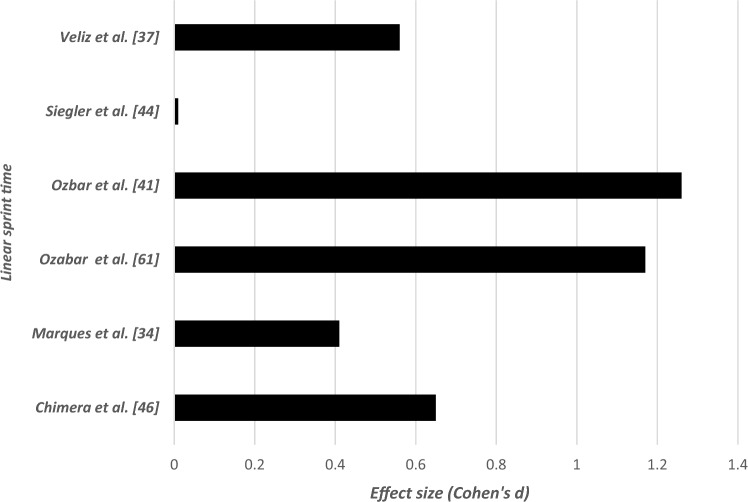
Summary of effect sizes of the identified studies on the effectiveness of RT on linear sprint performance in elite female athletes

**Fig. 4:**
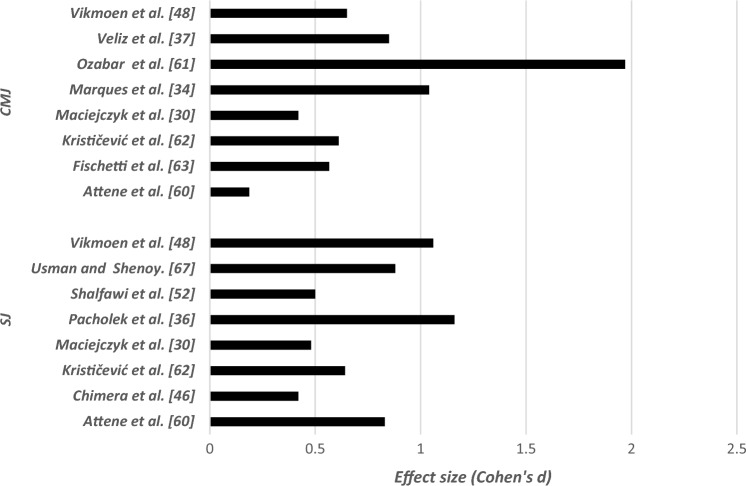
Summary of effect sizes of the identified studies on the effectiveness of RT on countermovement (CMJ) and squat jump (SJ) performances in elite female athletes

Table 4 summarizes the effects of long-term RT or combinations of RT with other strength-dominated exercise types on muscular fitness in elite female athletes. Irrespective of the type of RT or plyometric training and the applied exercise protocol (type of exercise, exercise duration, intensity), 23 out of 24 studies reported increases in power (e.g., maximal and mean power) (effect size [ES]: 0.23 < Cohen’s *d* < 1.83, small to large), strength (e.g., 1RM) (ES: 0.15 < *d* < 6.80, small to very large) (Fig. 2), speed (e.g., linear sprint times) (ES: 0.01 < *d* < 1.26, small to large) (Fig. 3), and jumping (e.g., countermovement jump [CMJ], squat jump [SJ]) (ES: 0.02 < *d* < 1.04, small to large) (Fig. 4). Only the study of Maciejczyk et al. [30] observed no training-induced changes in maximal power (ES: 0.42 < *d* < 0.56, small) and the fatigue index (*d* = 0.03, trivial) after 4 weeks of plyometric training in soccer players. The tested players of this study increased their performance in the squat jump (*d* = 0.48, small) and the Illinois Agility Test (*d* = 0.7, large). In eight out of nine studies pertaining to combined training [31–38], significant increases were found for measures of maximal strength, muscle power, jump, and sprint performance (ES: 0.08 < *d* < 2.41, small to very large). Only one study by Grieco et al. [39] observed no training-induced changes in the maximal isometric knee flexor and extensor strength (ES: 0.13 < *d* < 0.26, trivial to small) after 10 weeks of combined resistance, plyometric, and agility training in soccer players.

**Table 4:** Effects of long-term RT on measures of strength and proxies of muscular power in elite female athletes

Study	Participants (number/age)	Strength performances (1RM jump performances…)			Effect size	Data		%
						Before	After	
Attene et al. [60]	18/14.83 ± 0.92	*CMJ*			0.83	26.94 ± 3.62	29.99 ± 3.65	11.30
		↑ power (w/kg)			0.40	24.52 ± 7.35	27.47 ± 7.19	15.40
		↑ strength (n/kg)			0.75	20.22 ± 2.88	22.29 ± 2.60	+ 2.3
		↑ speed (cm/s)			0.64	149 ± 31.06	168 ± 28.03	12.75
		*SJ*			0.18	22.71 ± 3.24	26.21 ± 3.55	15.4
		↑ power (w/kg)			0.51	29.64 ± 4.14	31.77 ± 4.13	7.18
		↑ max power (w/kg)			0.15	31.59 ± 3.87	32.21 ± 4.21	2.25
		↑ strength (n/kg)			0.43	20.76 ± 4.53	22.44 ± 2.98	8.09
		↑ speed (cm/s)			0.93	183 ± 15.60	197 ± 14.39	7.65
Ness et al. [49]	17/18.8 ± 0.9	↑ isometric hip strength	External rotation	D	1	11.2 ± 2	13.6 ± 2.9	21.42
				ND	0.87	12.4 ± 1.9	14.4 ± 2.7	16.12
		↓ isometric hip strength		ND	0.12	35.2 ± 9.7	34.0 ± 9.8	− 3.40
		↑ lower extremity dynamic balance	D Limb	Composite	0.59	88.2 ± 6.3	91.8 ± 5.6	4.08
				Anterior	0.4	71.8 ± 5.1	73.9 ± 5.3	1.67
				Posterolateral	0.62	94.2 ± 8.3	99.2 ± 7.7	5.30
				Posteromedial	0.37	100.0 ± 9.5	103.0 ± 6.8	3
			ND Limb	Composite	0.71	86.9 ± 6.6	91.7 ± 6.9	5.52
				Anterior	0.66	70.3 ± 4.9	73.7 ± 5.2	4.83
				Posterolateral	0.85	94.8 ± 9.9	101.8 ± 6.8	7.38
				Posteromedial	0.31	100.6 ± 7.0	102.8 ± 7.9	2.18
Grieco et al. [39]	15/19.0 ± 0.7	↔ maximal isometric strength of knee flexion and extension	Knee flexion left		0.24	111.3 ± 27.5	118.3 ± 30.1	5.71
			Knee extension right		0.07	188.1 ± 42.6	184.8 ± 44.5	− 1.74
Cherni et al. [45]	15/20.9 ± 2.4	*Sprint times*						
		↔ 10 m (s)			0.65	2.15 ± 0.14	2.07 ± 0.10	
		↔ 20 m (s)			0.36	3.63 ± 0.21	3.56 ± 0.17	
		↔ 30 m (s)			0.43	5.11 ± 0.30	4.99 ± 0.25	
		↓ ability to change direction: test(s)			0.85	11.69 ± 0.59	11.23 ± 0.48	− 3.93
		↔ SJ height (cm)			0.24	33.9 ± 4.6	37.3 ± 4.8	
		↔ CMJ height (cm)			0.76	36.0 ± 4.8	37.2 ± 5.1	
		*SJ*						
		↑ RMS.VL (%)			0.94	91.57 ± 12.10	99.69 ± 0.55	8.12
		↑ RMS.RF (%)			0.77	98.16 ± 3.01	99.81 ± 0.35	1.65
		*CMJ*						
Chimera et al. [46]	9/20 ± 2	↑ vertical jump			0.42	17.89 ± 2.29	18.89 ± 2.45	5.58
		↓ sprint speed			0.65	7.21 ± 0.31	7.00 ± 0.33	2.91
		↓ seated hamstrings curl ↑ stiff-legged deadlift (kg)			1.93 1.79	33.8 ± 3.6 28.7 ± 3.9	39.2 ± 1.6 35.2 ± 3.3	15.97 22.64
Fischetti et al. [63]	14/26.5 ± 6.9	↑ CMJ			0.56	33.6 ± 5.5	36.8 ± 5.8	9.7%
		↓ T—Test (sec)			1	8.8 ± 0.3	8.5 ± 0.3	− 3.4%
Krističević et al. [62]	52/15.4 ± 1.32	↑ SJ			0.64	21.80 ± 4.22	24.28 ± 3.48	11.37
		↑ CMJ			0.61	28.08 ± 4.83	30.72 ± 3.74	9.40
Losnegard et al. [47]	3/21.3 ± 5.1	↑1RM strength in a seated pull-down						19 ± 2
		↑ 1RM strength in half squat						12 ± 2
		↑ *V*O_2_max relative to body mass during treadmill skate roller skiing						7 ± 1
Maciejczyk et al. [30]	17/21 ± 3	↑ SJ			0.48	26.23 ± 5.14	28.63 ± 4.76	4.16
		↑ Illinois Agility Test (s)			0.7	16.8 ± 0.88	16.2 ± 0.84	− 3.57
		↑ CMJ			0.42	28.11 ± 4.56	29.93 ± 5.01	6.47
Jones et al. [51]	49/75							
	*n* = 16	↑ 1RM bench						18
		↑ 1RM squat						18.9
		↑ VJ						7
		↑ SLJ (standing long jump)						5.6
	*n* = 21	↑ 1RM bench						2.7
		↑ 1RM squat						5.8
	*n* = 12	↑ 1RM bench						8.6
		↑ 1RM squat						8
Martel et al. [35]	10/15 ± 1	↑ vertical jump (cm)	VJ after 2 wk ↑ VJ after 4 wk ↑ VJ after 6 wk		0.06 0.19 0.80	33.4 ± 4.7	3.1 ± 4.7 34.4 ± 5.6 37.1 ± 4.5	2.99 11.07
		↑ concentric peak torque	*Dominant leg*					
			Knee flexion 60°s^−1^		0.79	69 ± 13	79 ± 12	14.49
			Knee extension 60°s^−1^		0.44	108 ± 29	120 ± 25	11.11
			Knee flexion 180° s^−1^		0.68	48 ± 13	56 ± 10	16.66
			Knee extension 180°s^−1^		0.41	61 ± 17	69 ± 21	13.11
			Non-dominant leg					
			Knee flexion 60°s^−1^		0.79	67 ± 16	79 ± 14	17.91
			Knee extension 60°s^−1^		0.62	97 ± 24	113 ± 27	16.49
			Knee flexion 180° s^−1^		0.33	56 ± 25	63 ± 16	12.5
			Knee extension 180°s^−1^		1.10	52 ± 16	72 ± 2	38.46
Marques et al. [33]	10/25.3 ± 1.3	↑ in upper body strength (á 4RM-BP)			2.2	40 ± 2.8	47 ± 3.5	17.5
		↑ ball throwing distance			1.06	720 ± 67	816 ± 109	13.33
		↑ lower body strength (4RM-PS)			0.96	92 ± 11.1	104 ± 13.6	13.04
		↑ unloaded and loaded CMJ	Unloaded		0.21	34.22 ± 5.9	35.56 ± 6.28	3.91
			Loaded (10 kg)		0.58	26.41 ± 3.83	28.95 ± 4.8	9.61
			Loaded (20 kg)		0.68	21.82 ± 2.89	24.07 ± 3.61	10.31
			Loaded (30 kg)		0.73	18.70 ± 2.64	21.07 ± 3.69	12.67
Marques et al. [34]	5F/16.8 ± 0.8	↑ CMJ			1.04	26.4 ± 2.6	30.2 ± 2.9	14.39
		↑ 1RM – SQ kg			1.10	46.4 ± 7.4	53.6 ± 6.0	15.51
		↑ 1RM – BP kg			1.04	35.8 ± 5.0	41.0 ± 5.7	14.52
		↑ pull-up rep			1.11	2.5 ± 1.9	6.0 ± 4.2	140
		↓ 50-m (s)			0.41	30.71 ± 1.93	29.73 ± 1.90	− 3.19
Montalvo-Pérez et al. [13]	TRT *n* = 8/26 ± 7	↑ squat 1RM			1.67	48 ± 13	65 ± 6	35.41
		↑ squat MMP (W)			0.03	285 ± 98	362 ± 71	27.01
		↑ squat MMP [W/lower body muscle mass (kg)]			0.00	20 ± 5	27 ± 5	35
		↑ hip thrust 1RM (kg)			0.33	62 ± 19	84 ± 15	35.48
	VBRT *n* = 9/24.6 ± 1.3	↑ hip thrust MMP (W)			0.33	278 ± 98	363 ± 71	30.57
		↑ hip thrust MMP [W/lower body muscle mass (kg)]			0.24	20 ± 4	27 ± 4	35
		↑ split squat 1RM (kg)			0.050	43 ± 10	59 ± 11	
		↑ split squat MMP (W)			0.020	228 ± 74	328 ± 82	
		↑ split squat MMP [W/lower body muscle mass (kg)]			0.004	17 ± 5	24 ± 4	
Myer et al. [64]	8/15.9 ± 0.8	↑ 1RM BP			0.861	30.9 ± 5.8	36.3 ± 6.7	18.44
		↑1RM hang clean			2.776	27.5 ± 3.5	40.3 ± 5.5	46.54
		↑ 1RM parallel squat			4.913	44.0 ± 5.8	81.2 ± 9.0	84.2
Nunes et al. [40]	12/26.2 ± 3.9	↑ 1RM	BP		0.14	52.0 ± 5.4	60.0 ± 5.7	15.38
			Half-squat		22.23	70.0 ± 4.7	81.5 ± 6.0	16.42
			Biceps curl		10.48	27.5 ± 2.5	33.0 ± 2.1	20
		vertical jump	↑ left leg		0.58	37.4 ± 4.3	39.6 ± 3.1	4.94
			↑ both legs		0.64	48.9 ± 4.2	51.3 ± 3.2	4.90
		↑ repetitions performed with a 50% 1RM	BP		0.3	18.5 ± 2.1	26.0 ± 3.5	40.54
			half-squat		2.39	41.0 ± 3.5	49.5 ± 3.6	20.73
			biceps curl		2.88	25.5 ± 2.1	32.0 ± 2.4	25.49
Ozabar et al. [55]	9/18.2 ± 2.3 (15–22)	↑ triple jump	D leg		1.54	4.9 ± 0.5	5.6 ± 0.4	14.28
			ND leg		1.56	4.9 ± 0.6	5.7 ± 0.4	16.32
		↑ standing broad jump			0.67	182.8 ± 13.5	192.3 ± 14.6	5.19
		↑ CMJ			1.97	39.8 ± 4.5	46.8 ± 2.2	17.58
		↑ peak power			0.63	3480.0 ± 643.2	3855.2 ± 536.6	10.7
		↓ 20-m sprint time			1.17	3.7 ± 0.3	3.4 ± 0.2	8.10
Ozbar [41]	10/19.4 ± 1.6 (18–22)	↑ CMJ			3.58	40.1 ± 1.9	48.6 ± 1.6	21.19
		↑ SBJ			1.54	182.5 ± 12.4	193.5 ± 12.6	6.02
		↑ PP			1.20	3438.9 ± 497.3	3894.5 ± 470.7	13.24
		↑ kicking speed	D		1.34	83.2 ± 5.9	91.4 ± 7.7	9.85
			ND		1.06	71.0 ± 4.2	79.5 ± 5.3	11.97
		↓ 10-m sprint time			1.26	2.3 ± 0.7	2.0 ± 0.1	13.04
		↓ 20-m sprint time			1.26	3.8 ± 0.3	3.4 ± 0.2	10.52
		↓ 30-m sprint time			0.15	5.3 ± 0.4	4.8 ± 0.2	
Pacholek et al. [36]	13/20.2 ± 3.3	↔ time of the shuttle run			0.500	18.22 ± 0.56	18.47 ± 0.43	
		↑ explosive power in lower limbs			1.16	45.9 ± 8.7	55.9 ± 8.5	21.78
		↑ vertical jump height			1.16	29.4 ± 3.9	34.7 ± 5.1	18.02
		↑1RM-BHS			0.63	60 ± 8.55	65.8 ± 9.77	9.66
		↑1RM-BBP			0.349	35 ± 6.03	37 ± 5.41	5.71
		↓ time of the shuttle run			0.9	18.49 ± 0.51	18.05 ± 0.46	− 2.37
		↓ explosive power in lower limbs			0.83	59.7 ± 10.6	51.9 ± 8	13.06
		↓ Vertical jump			0.61	36.7 ± 4.9	33.8 ± 4.6	7.90
		↑ 1RM-BHS			0.85	59.2 ± 10.35	68.8 ± 12.11	16.21
		↑ 1RM-BP			0.84	34 ± 4.87	38 ± 4.65	1.76
		↑ 1RM-BBP			0.215	37 ± 4.62	38 ± 4.65	2.70
Pereira et al. [65]	10/14.0	↑ upper body medicine ball distance						3
		↑ throwing volleyball distance						19.6
		↑ CMJ						20.1
Shalfawi et al. [52]	19.4 ± 4.4	↑ Beep-test			1.0	9.7 ± 1.3	10.9 ± 1.2	12.37
		↑ SJ performance			0.5	25.9 ± 2.7	27.5 ± 4.1	6.17
Siegler et al. [44]	17/16.5 ± 0.9	↑ LIST (seconds to failure)			2.424	646.00 ± 167.47	1040.00 ± 157.33	60.99
		↓ 20-m sprint (s)			0.010	3.00 ± 0.15	2.90 ± 0.13	3.33
Skattebo et al. [43]	9/18 ± 1	↑ 1RM in seated pull-down (upper body strength)						
Usman and Shenoy [67]	30/19.2 ± 0.8	↑ VJH	2 weeks		1.13	42.19 ± 0.85	43.6 ± 1.76	3.34
			4 weeks		1.13	43.61 ± 1.76	45.90 ± 2.26	5.25
			6 weeks		0.82	45.90 ± 2.26	47.76 ± 2.26	4.05
			8 weeks		1.06	47.76 ± 2.49	50.08 ± 1.83	4.87
Newton et al. [50]	14:20.0 ± 1.2	↑ jump distance (cm)	Mid-season (7 weeks) End season (11 weeks) ES (start–end)		0.605 0.568 0.035	61.2 ± 5.6 57.9 ± 5.3	57.9 ± 5.3 61.0 ± 5.6	5.4 5.3
		↑ absolute jump height (cm)	Mid-season (7 weeks) End season (11 weeks) ES (start–end)		0.320 0.284 0.028	291.9 ± 10.4 291.6 ± 10.2	294.6 ± 10.2 294.6 ± 10.9	1.11
		loaded jump squat testing ↑ average force (N)	Mid-season (7 weeks) End season (11 weeks) ES (start–end)		0.160 0.620 0.871	1.68 ± 1.93 11.72 ± 2.73	1.72 ± 2.73 1.89 ± 2.81	9.9 12.4
		↑ average power (W)	Mid-season (7 weeks) End season (11 weeks) ES (start–end)		0.234 0.564 0.826	2.28 ± 289 2.35 ± 3.40	2.359 ± 3.40 2.5 ± 3.71	8.8 12
		jump squat testing ↑ peak force (N)	mid-season (7 weeks) end season (11 weeks) ES (start–end)		0.520 0.062 0.575	1.474 ± 145 1.549 ± 143	1.549 ± 143 1.558 ± 147	5.7
		CMJ testing ↑ peak force (N)	Mid-season (7 weeks) End season (11 weeks) ES (start–end)		0.070 0.565 0.635	1.48 ± 1.57 1.49 ± 1.55	1.49 ± 1.55 1.57 ± 1.38	5.6 6.3
		↑ peak power (W)	mid-season (7 weeks) end season (11 weeks) ES (start–end)		0.699 1.000	3.05 ± 3.68 2.83 ± 2.57	2.83 ± 2.57 3.13 ± 3.39	7.3 10.6
		30-cm drop jump testing peak power (W)	Mid-season (7 weeks) End season (11 weeks) ES (start–end)		0.407 0.656 0.052	3.09 ± 3.84 2.95 ± 2.99	2.95 ± 2.99 3.21 ± 4.74	8.8
		60-cm drop jump testing average force (N)	Mid-season (7 weeks) End season (11 weeks) ES (start–end)		0.149 0.565 0.784	1.43 ± 1.04 1.45 ± 170	1.45 ± 1.70 1.55 ± 2.04	8.9
		↑ peak power (W)	Mid-season (7 weeks) End season (11 weeks) ES (start–end)		0.218 0.903 0.836	2.93 ± 2.09 2.87 ± 3.06	2.87 ± 3.06 3.15 ± 3.18	9.8 7.7
Veliz et al. [37]	11/26.4 ± 4.3	↑ height in the water jump			1.2	38.41 ± 4.52	43.02 ± 3.21	12.00
		↑ CMJ			0.85	28.63 ± 2.93	31.11 ± 2.83	8.66
		↑ peak power			0.33	2877.33 ± 145.7	3027.75 ± 161.86	5.2
		↑ full squat 1RM			2.41	60.88 ± 5.34	73.66 ± 5.67	20.99
		↑ relative full squat			1.01	0.73 ± 0.19	0.92 ± 0.18	26.02
		↑ water polo throwing speed			3.44	50.11 ± 1.04	53.55 ± 1.11	6.86
		↓ 20 m swim time			0.56	12.93 ± 0.32	12.76 ± 0.34	− 1.3
Vikmoen et al. [48]	11	↑ 1RM (%)			3.2			40.4 ± 14.7
		↑ SJ (%)			1.06			8.9 ± 6.8
		↑ CMJ			0.65			5.9 ± 6
Vikmoen et al. [42]	11/31.5 ± 8.0	↑ 1RM one-legged leg press						39 ± 19
		↑ maximal isometric torque						12 ± 11
		↑ 6RM load (kg)						39 ± 11
		↑ SJ						24.3 ± 6.0
		↑ CMJ						25.6 ± 4.2
		↑ peak torque at 240°·s^−1^						8 ± 5
Vikmoen et al. [11]	11	↑ 1RM			2.4			45 ± 22
		↑ 5-min all-out tests			0.62			7.0 ± 4.5
Voelzke et al. [38]	8/26.0 ± 7.0	↑ SJ						2.3
		↑ three-step reach height			0.03	292.3 (39)	293.5 (41)	1.6
		↑ CMJ						3.8
		↑ drop jump			0.10	1.09 (0.69)	1.16 (0.59)	6.4
		↑ three-step reach height			0.12	306.5 (39)	311.5 (41)	1.63
		↓ 15 m lateral				5.38 (978)	5.18 (1.27)	− 3.71
Wilkerson et al. [74]	11/19 ± 1.4	Isokinetic peak-torque	↑ 60°·s^−1^ hamstrings peak torque (n/m)		0.37	90.81 ± 17.91	98.12 ± 20.91	8.80
		↔ isokinetic peak-torque ratios	60°·s^−1^ quadriceps/body weight		1.20	76.61 ± 9.95	88.28 ± 9.41	15.25
			60°·s^−1^ hamstrings/body weight		1.15	40.70 ± 4.84	47.51 ± 6.79	16.37
			300°·s^−1^ quadriceps/body weight		1.46	39.45 ± 5.66	48.10 ± 6.15	21.92
			300°·s^−1^ hamstrings/body weight		1.23	27.05 ± 5.68	32.73 ± 3.16	20.65

*BBP* barbell bench press, *BHS* barbell half squat, *BP* bench press, *CG* control group, *CMJ* countermovement jump, *D* dominant, *ES* effect size, *IG* intervention group, *LIST* shuttle test, *Max* maximum, *MMP* maximum mean power output, *N* sample size, *ND* non-dominant, *PP* peak power, *RE* resistance endurance training, *Reps* repetitions, *RF* rectus femoris, *RM* the maximum load an individual can lift under standardized conditions, *RMS* root mean square, *RT* resistance training, *SBJ* standing broad jump, *SJ* squat jump, *SQ* full squat, *TRT* traditional resistance training, *VBRT* velocity-based resistance training, *VJH* vertical jump height, *VL* vastus lateralis, *VM* vastus medialis

### Effects of Long-Term Resistance Training on Body Composition and Muscle Morphology in Elite Female Athletes

Eleven studies investigated the long-term effects of RT or combinations of RT with other strength-dominated exercise types on body composition and muscle morphology in elite female athletes (Table 5). Four out of six studies observed no changes in body mass or percentage of body fat (ES: 0.026 < *d* < 0.49, small to medium) after 10 weeks of resistance, plyometric, and agility training in soccer players [41], or after 12 weeks of RT in basketball players [40], or 10 weeks of a plyometric training program in elite female soccer players [41], and/or after 11 weeks of RT in endurance-trained athletes [42]. However, Skattebo et al. [43] reported an increase in body mass (2.5 ± 1.2%) after 10 weeks of RT in well trained young cross-country skiers. Siegler et al. [44] reported a decrease in mass fat (− 1.40 ± 1.47 kg, ES = 0.313) of 17 female soccer players who completed 10 weeks of in-season, plyometric, resistive training and a high-intensity anaerobic program.

**Table 5:** Effects of long-term RT on measures of body composition and muscle morphology in elite female athletes

Study	Participants (number/age)	Intervention			Muscle morphology (e.g., muscle mass, muscle size, muscle fiber…) and body composition (body mass index, body mass, fat mass, and muscle mass…)					
		Duration	Frequency per week	Volume			Effect size	Before	After	% change
Grieco et al. [39]	15/19.0 ± 0.7	10 weeks of strength-plyometric-agility training program	2	60 min 3 sets of 9–10 exercises	↔ body mass (kg)		0.026	59.9 ± 6.7	60.1 ± 8.4	0.33
					↔ body fat (%)		0.492	18.2 ± 1.7	19.4 ± 3.0	6.59
Cherni et al. [45]	15/20.9 ± 2.6	8 weeks of plyometric training: sprinting, jumping, agility, peak power, average power, and associated neuromuscular adaptations	2		Lower-limb muscle volumes	↑ leg muscle volume (L)	0.322	9.2 ± 1.5	9.7 ± 1.6	5.43
						↑ thigh muscle volume (L)	0.384	6.1 ± 1.3	6.6 ± 1.3	8.19
					CSA	↑ mean thigh CSA (cm^2^)	0.211	155 ± 26	160 ± 21	3.22
						↑ maximal thigh CSA (cm^2^)	0.654	226 ± 30	247 ± 34	8.50
					↓ body fat %		0.344	23.5 ± 4.3	22.0 ± 4.4	− 6.38
Chimera et al. [46]	9/20 ± 2	6 weeks of plyometric training	2 days per week	20–30 min each session	Auadriceps: hamstring coactivation	↓ preparatory	0.893	0.66 ± 0.29	0.43 ± 0.22	− 34.48
						↓ reactive	0.128	1.39 ± 0.38	1.35 ± 0.22	− 2.87
					Adductor: abductor coactivation	↑ preparatory	0.59	0.48 ± 0.12	1.02 ± 0.46	112.5
						active	0	1.12 ± 0.25	1.12 ± 0.29	
Losnegard et al. [47]	3/21.3 ± 5.1	12 weeks RT			↑ CSA in m. triceps brachii					5.5 ± 2.1
					↑ upper body LBM					3.0 ± 1.1
Luteberget et al. [32]	8/20.4 ± 3.1	10 weeks of resisted sprint training, 2 sessions per week with an additional weight of 12.4% ± 0.2% of body mass			↑ in fascicle length		0.46	7.7 ± 0.6	8.0 ± 0.5	3.89
					↔ muscle thickness		0.1	2.34 ± 0.24	2.37 ± 0.26	1.28
					↔ pennation angle		0	17.8 ± 2.5	17.8 ± 2.1	
	6/23.1 ± 3.9	10 weeks of traditional sprint training, 2 sessions per week			↑ in fascicle length		0.26	7.7 ± 1.3	8.1 ± 1.3	5.19
					↔ muscle thickness		0.07	2.39 ± 0.30	2.37 ± 0.31	
					↓ pennation angle		0.38	18.3 ± 2.5	17.3 ± 3.0	− 5.46
Nunes et al. [40]	12/26.2 ± 3.9	RT: 12 weeks, 10–12 training sessions per week, 3–4 sets with 15–20 repetition maximum (RM) loads strength for 2 weeks: 3–6 sets with 1–10RM loads and power for 2 weeks: 3–6 sets of 6–10 reps with 30–50% 1RM loads			↓ ∑ skinfolds (mm)		0.29	72.8 ± 21.6	66.8 ± 19.7	− 8.24
					↔ body mass (kg)		0.007	82.2 ± 13.1	82.3 ± 12.6	
					↔ triceps (mm)		0.102	16.8 ± 6.9	16.1 ± 6.7	
					↔ subscapular (mm)		0.139	16.3 ± 5.9	15.5 ± 5.6	
					↔ abdominal (mm)		0.131	22.4 ± 6.3	21.6 ± 5.9	
					↔ body fat (%)		0.196	16.9 ± 3.1	16.3 ± 3.0	
					↔ supra iliac (mm)		0.394	17.4 ± 4.6	15.7 ± 4.0	
Ozbar [41]	10/19.4 ± 1.6 (18–22)	10 weeks of plyometric training 2 times per week			≈ height, cm		0.101	163.6 ± 4.7	163.1 ± 5.1	
					≈ mass, kg		0.444	58.0 ± 6.6	55.3 ± 5.5	
					≈ body mass index, kg.m^−2^		0.390	21.7 ± 2.2	20.8 ± 2.4	
Siegler et al. [44]	17/16.5 ± 0.9	10-week in-season plyometric, resistive training and high-intensity anaerobic program			↑ fat-free mass		0.172	49.33 ± 6.37	50.48 ± 6.92	2.33
		and high-intensity anaerobic program			↓ fat mass		0.313	12.13 ± 4.66	10.73 ± 4.26	− 11.35
Skattebo et al. [43]	9/18 ± 1	10 weeks of heavy strength training			↑ body mass					2.5 ± 1.2
Vikmoen et al. [48]	11	RT, twice a week for 11 weeks, four leg exercises (3 × 4–10 RM)			↑ muscle fibers CSA type I (m. vastus lateralis) (%)				13.2 ± 6.8	
					↑muscle fibers CSA type II (m. vastus lateralis) (%)				30.8 ± 19.6	
					↔ stiffness of the patellar tendon		0.455	2752 ± 402	2483 ± 733	
					↔ young’s Modulus of the patellar tendon		0.632	1038 ± 194	925 ± 162	
					↑ mean CSA of the patellar tendon		0.471	65.9 ± 7.1	69.2 ± 6.9	5.00
Vikmoen et al. [42]	11	11 weeks of RT consisting of four lower body exercises (3 × 4–10 RM) twice a week			≈ body mass		0.129	62.4 ± 5.2	63.1 ± 5.6	
					↓ muscle fiber positive type: II A, IIX		0.13			9 ± 7
					↑ muscle fiber IIA		1.03	39 ± 13%	51 ± 10%	30.76
					↓ leg LM		1.69			3.1 ± 4.0

*CG* control group, *CSA* cross-sectional area, *EMS* + *P* electromyostimulation and plyometric training, *IG* intervention group, *LBM* lean body mass, *LM leg* lean mass in the legs, *Max* maximum, *N* sample size, *RE* resistance endurance training, *Reps* repetition, *RM* the maximum load an individual can lift under standardized conditions, *RT* resistance training, *TRT* traditional resistance training

Five out of six studies [37, 42, 44–48] showed significant training-induced changes in muscle morphology (e.g., muscle thickness, muscle fiber cross-sectional area) (ES: 0.23 < *d* < 3.21, small to very large). However, one study could not detect any changes in muscle morphology (i.e., muscle thickness, pennation angle) [32].

## Discussion

To the best of our knowledge, this is the first systematic review that reports the long-term effects of RT or combinations of RT with other strength-dominated exercise types on muscular fitness, muscle morphology, and body composition in female elite athletes. Findings from this systematic review show that RT alone or in combination with other strength-dominated exercise types leads to significant changes in measures of muscle morphology, muscle power, strength, speed, and jump performance in elite female athletes. The question pertaining to the optimal programming parameters (e.g., frequency, intensity, volume) is still open and should be addressed in future studies.

### Muscular Strength Improvements With Resistance Training

Despite the general agreement that prescribing various types of RT, alone or combined with other exercise types, could improve parameters, especially strength, power, linear sprint speed, and jump performance in athletes [22], only a few studies have explored whether such interventions and types of RT were beneficial for elite female athletes.

While RT has previously been shown to be effective in improving measures of muscle strength [13, 31, 37, 49, 50], power [13, 31, 37, 50], and speed [31, 50], such results were obtained by including studies with intervention durations ranging from 4 to 12 weeks. Interestingly, we observed that RT induced large gains in measures of maximal strength (i.e., 1RM, leg press, half squat, and bench pull) in elite females. The maximum strength gain ranged from 8% to 18.9% in response to heavy resistance (lower body push, upper body push, and circuit of abdominal and low back exercises) after 8–12 weeks [50, 51].

Twelve analyzed articles evaluated the effects of RT on 1RM performance (9.66–45%) [13, 31, 32, 34, 36, 37, 40, 42, 43, 47, 48, 51, 52]. Changes in this variable have been reported in several muscle strength tests, such as the squat (5.8–18.9%), bench press (2.7–18.0%), pull-down (13.3–13.6%), and leg press exercise (16.4–44.3%) at full or partial ranges of motion. Larger training-induced gains were found if the training program mimicked the requirements of the tested outcome [53] and was therefore in accordance with the principle of training specificity.

Initial training status plays an important role in the rate of progression during RT. Training status reflects a continuum of adaptations to RT such that level of fitness, training experience, and genetic endowment each contribute. Quantification of strength gains appears to occur within a few months of training. Changes in strength are most pronounced early in training when the 'window of adaptation' is greatest [54]. Short-term studies (i.e., 6–24 weeks) have shown that the majority of strength gains occur within the first 4–8 weeks [55]. A limited number of studies have examined different models of progression during long-term RT. However, little is known about the adaptations and improvements in strength in response to prolonged training in elite female athletes. The rate of strength gains varies considerably between untrained and trained individuals, with trained individuals showing much slower rates of improvement [56]. A review of the literature suggests that muscle strength increases by approximately 40% in 'untrained', 20% in 'moderately trained', 16% in 'trained', 10% in 'advanced', and 2% in 'elite' individuals over periods ranging from 4 weeks to 2 years [57].

Therefore, longer-term RT studies are needed to determine the upper limits of the dose–response relationship between training volume and muscular adaptations [58]. Discrepancies between studies remain unclear, but it appears that the dose–response relationship is more pronounced in resistance-trained individuals. It is not clear whether regular training at maximal loads promotes a superior strength-related response to this metric and, if so, how much loading should be incorporated into a comprehensive training program to optimize results. In contrast, trained individuals may require a greater stimulus (e.g. heavier load or greater intensity of effort) to continue to make positive adaptations [58].

Research in highly trained individuals on this topic is lacking, but it seems likely that continued improvements in maximum strength will become increasingly dependent on training closer to an individual's 1RM as they approach their genetic ceiling. Indeed, there is evidence to suggest that the principle of specificity (also known as a specific adaptation to imposed demands) becomes more relevant as the level of training experience increases [59]. Further study is warranted in elite athletes to better understand how training experience impacts the acquisition of strength with respect to the magnitude of the load. It is not clear whether regular training with maximal loads promotes a superior strength-related response on this metric and, if so, how much loading should be integrated into a comprehensive training program to optimize results.

Many sports performance coaches use periodized programs when preparing athletes for muscular anatomical adaptations prior to competition. Thus, future studies should evaluate muscular adaptations in periodized programs, particularly in trained athletes over a long duration. This type of study would simulate adaptations that occur as athletes progress through various phases of competition, such as in-season, post-season, and off-season.

### Muscular Power Improvements with Resistance Training

With regards to the outcome of muscle power, our analyses including 15 studies revealed larger training-induced adaptations following plyometric training compared with RT in elite females. Examples of plyometric exercises included explosive jumps, hops, bounds, and skips. Possibly owing to the demand for higher force production at higher velocities, plyometric training has been shown to exhibit a large advantage over RT for improvements in power (7.2–15.4%) [41, 60], speed (2.9–13.0%) [41, 46, 60, 61], and jumping measures (CMJ: 5.2–20.1%; SJ: 4.2–15.4% and drop jump [DJ]: 5.8–18.4%) [35, 46, 51, 60–68]. Plyometric training may elicit adaptations in a wider range of physical qualities across the strength and power continuum in comparison with RT. Plyometric training appeared to be more effective in improving jump performance, whereas free-weight RT was more advantageous in improving maximum strength (where the stretch reflex is not involved). Typically, plyometric training is conducted over a period of several days or weeks (6–12 weeks), at a training frequency of 1–3 sessions per week, and a maximal to near-maximal intensity [68].

Despite the available evidence on the effectiveness of RT in female athletes, there is a need for further research [69–71]. This is because of the small number of studies (in comparison with the amount of research conducted on male athletes) and the heterogeneity of the applied study protocols which vary in duration, frequency, intensity, and volume.

Ramirez-Campillo et al. [71] indicated that, in future research, specific dose–response relationships following plyometric training should be identified. An interesting direction would be to determine the minimum duration of plyometric training in highly trained women. Meta-analyses conducted to date indicate that longer training durations (> 10 weeks) yield greater improvement in jump performance [67], and shortening the duration of plyometric training may be crucial in developing jumping performances.

It has been shown that plyometric exercise can also be effective when performed for only 4 weeks, instead of the typically applied periods of 6–12 weeks. Due to the usually short pre-season and long regular soccer season, coaches can prepare their training plan better and more effectively with shorter durations of plyometric training.

When comparing improvements in strength from RT and plyometrics, it may be suggested that RT results in superior strength gains. Despite these findings, there is no reported consensus highlighting the magnitude of differences between RT and plyometrics.

On the other hand, to establish practical applications and guidelines for researchers and practitioners employing and investigating these training methods, current recommendations for exercise prescription suggest combined RT because the benefits may provide an overall synergistic effect, and each intervention has overlapping and unique benefits [72]. Ten studies dealing with combined training, such as resistance, plyometric, and agility training; resistance and sprint training; resistance and power training; resistance and plyometric training [31–39, 44], showed significant increases in maximal strength (lower and upper body strength, full squat 1RM), peak power, jump performance (squat jump and CMJ), and linear sprint speed (5-, 10-, 20-m and the Loughborough Intermittent Shuttle Test (LIST).

Several studies [33, 34, 37, 38, 44] seem to indicate that a combination of resistance and plyometric training is likely to elicit the greatest improvements (i.e., greater improvements in jump height due to plyometrics, as well as greater improvements in strength and sprint measures due to RT), especially if multiple outcome parameters are tested, and it is therefore recommended from a practical standpoint. Only one study [39] observed no change on the maximal isometric strength of knee flexion and extension (ES: 0.13 < Cohen’s d < 0.26, trivial to small) after 10 weeks of combined strength and plyometric agility training in female soccer players. The purpose of the noted study was to determine the extent to which an off-season combined resistance-plyometric-agility training program would affect $\dot{V}$O_2peak_ and running economy in collegiate female soccer players. Accordingly, a significant increase in $\dot{V}$O_2peak_ (10.5%) was found, in the absence of a consistently significant increase in the RE at 9 km/h, after a 10-week training program. Furthermore, there was no significant change in maximal isometric strength of knee flexion or extension, which was likely due to the lack of testing specificity when associating isometric measures with dynamic functional athletic performances [73]. Such a relationship has been previously reported by several authors [73, 74]; for instance, McKay et al. [20] found that absolute isometric peak torque was not correlated with jump height. These results revealed that plyometric/sprint training-related jumping improvements were not due to increased maximal knee extension torque production.

Some controversial results exist concerning the ‘transfer of training effect’ from different methods of RT programs to various athletic performance parameters. A specific strength training method needs to be chosen based on the variables to be influenced. Knowledge of the effects of each method is crucial for the success of training with respect to physical and sports performance and in terms of preventing injuries [70].

Specifically, Harris et al. [75] showed that the combination model of training was effective for improving most of the strength tests compared with models of training-based force or high power. The characteristics of the combination model are not the same in these studies, as they are a variable combination of different methods for developing strength abilities, using their advantages to achieve the best force gradient.

For athletes who have experienced long-term RT, their power may increase to a higher level. However, it becomes complicated to increase the sizes and strength of other muscles. Adding a chain or elastic band to the free weights or changing the state of the body movement can provide a new stimulus for the muscles and improve the coordination between the muscles in the fight against unfixed resistances, thus improving the development of strength [76]. Future research should refine the training load, such as distinguishing between different-level trainers, and the proportion of variable resistance in the total load.

### Body Composition Improvements with Resistance Training

Resistance training has several positive impacts on the bodies of athletes. The most obvious effect is related to the amount of muscle mass, which is represented by the muscle cross-sectional area. Adequately applied RT can lead to increases in maximal strength of > 20% after a 21-week training program [77–79]. A similar effect on muscle morphology (e.g., muscle thickness, muscle fiber cross-sectional area) has also been described for a RT program in females after 10–12 weeks of progressive RT [32, 47, 48].

Progressive RT in females can lead to a significant increase in strength and muscle mass. This may primarily be due to the observed adaptations in the central nervous system (efficient motor unit recruitment, firing frequency), especially during the first weeks of training [79, 80]. In this context, it is plausible that neural adaptations in female athletes are more pronounced during the first weeks of RT for upper-body muscles [81, 82] or lower-body muscles [83], resulting in higher relative strength gains.

Various types of RT may elicit acute and, in some cases, chronic hormonal changes, which appears to play an essential role in mediating hypertrophic signaling reactions [84]. The three most often studied hormones are insulin-like growth factor (IGF-1), testosterone, and growth hormone (GH). Accordingly, we regard these as practically and clinically relevant in terms of anabolic reactions and responses to RT [85, 86].

### Muscle Morphology Improvements with Resistance Training

Some studies [39, 84], while monitoring relative muscle adaptation to different types of training, have shown that the ratio of the cross-section of muscle and neural adaptation provides the best indicator of force–velocity fitness, which focuses on explosiveness, maximum power, and velocity strength. Many studies have shown that the most effective method of enhancement of muscle power is the application of plyometric and/or free weight training consisting of maximal, submaximal, and light weights, as well as a combination of these methods [85, 86].

### Study Limitations

The results of the current systematic review have some limitations to note. In fact, in the current review, a systematic review approach was used with no meta-analysis performed, which should be considered by future investigators. The scope and approach of our review were broad, which is reflected by the large variety of training and testing protocols and the parameters measured. Moreover, some studies do not precisely report the training program (e.g., frequency, the load used, recovery periods, etc.) and as such that may have influenced the measured performance or muscle morphology. The heterogeneity of the methodological approach between studies is also represented by the different collection methods (application of apparatus) used to measure the outcomes. There is also a lack of data on some muscle morphology (e.g., muscle thickness, muscle fiber cross-sectional area) in many studies. Finally, there is great contemporary interest in the role of reproductive hormonal changes across the menstrual cycle to impact a women's training adaption and/or performance [87]. We did not address this point in this review and chose to approach the topic more from a sex comparison perspective. However, we acknowledge the role of the menstrual cycle on the responses to RT in women is a viable topic in need of further study.

## Scientific Conclusion/Clinical and Practical Application

Findings from the current systematic review indicate that resistance or combinations of RT with other strength-dominated exercise types induce significant improvements in muscular fitness, speed, and jump performance in elite female athletes. However, the optimal dosage of RT intensity and duration necessary to produce the most effective results in this population remains unclear.

## Declarations

### Data Availability Statement

All data supporting the findings of this study are available in this published article.
